# Hidden Effects of Seed Quality Breeding on Germination in Oilseed Rape (*Brassica napus* L.)

**DOI:** 10.3389/fpls.2018.00419

**Published:** 2018-04-03

**Authors:** Sarah Hatzig, Frank Breuer, Nathalie Nesi, Sylvie Ducournau, Marie-Helene Wagner, Gunhild Leckband, Amine Abbadi, Rod J. Snowdon

**Affiliations:** ^1^Department of Plant Breeding, Justus-Liebig University of Giessen, Giessen, Germany; ^2^KWS SAAT SE, Einbeck, Germany; ^3^Institute for Genetics, Environment and Plant Protection, Le Rheu, France; ^4^Groupe d’Etude et de Contrôle des Variétés et des Semences, Beaucouzé, France; ^5^NPZ Innovation GmbH, Holtsee, Germany

**Keywords:** canola, rapeseed, glucosinolates, erucic acid, genome-wide association study, vigor, linkage drag, genetic bottleneck

## Abstract

Intense selection for specific seed qualities in winter oilseed rape breeding has had an inadvertent negative influence on seed germination performance. In a panel of 215 diverse winter oilseed rape varieties spanning over 50 years of breeding progress in winter-type rapeseed, we found that low seed erucic acid content and reduced seed glucosinolate content were significantly related with prolonged germination time. Genome-wide association mapping revealed that this relationship is caused by linkage drag between important loci for seed quality and germination traits. One QTL for mean germination time on chromosome A09 co-localized with significant but minor QTL for both seed erucic acid and seed glucosinolate content. This suggested either potential pleiotropy or close linkage of minor factors influencing all three traits. Therefore, a reduction in germination performance may be due to inadvertent co-selection of genetic variants associated with 00 seed quality that have a negative influence on germination. Our results suggest that marker-assisted selection of positive alleles for mean germination time within the modern quality pool can help breeders to maintain maximal germination capacity in new 00-quality oilseed rape cultivars.

## Introduction

Oilseed rape (canola, rapeseed: *Brassica napus* L.) is today one of the most important oil crops worldwide, with a current global oil production of over 24 Mt^1^. This success is attributable to intensive breeding for seed quality traits during the last half century. The first milestone in rapeseed quality breeding was achieved by the removal of EA from the seed oil, resulting in high-quality oil with high levels of desirable unsaturated fatty acids. A further breakthrough came with the establishment of cultivars with low seed GSL content, allowing the use of rapeseed press residues as protein rich fodder in animal nutrition. However, the success of quality breeding in establishing a leading global crop was associated with significant genetic bottlenecks that have greatly diminished genetic diversity in modern breeding pools (Hasan et al., 2006; Bus et al., 2011; Qian et al., 2014). Both seed quality traits – low EA and low GSL content – originated from single genetic resources (reviewed by Friedt and Snowdon, 2009). Low EA derived from the German spring rapeseed variety “Liho,” which carried mutations in two *Bna.FAE1* homologs, while low GSL content was first identified in the Polish spring rapeseed cultivar “Bronowski.” Large-scale backcrossing programs facilitated the introgression of these traits into all the different ecogeographical forms of oilseed *B. napus*, initially in spring-sown germplasm in Canada and subsequently into winter-type germplasm in Europe. The first double-low (00) winter oilseed rape cultivar, “Librador,” was released in Germany in 1981.

Narrow genetic diversity considerably restricts breeding progress, limiting adaptation potential to biotic and abiotic stress factors and restricting the development of heterotic pools for hybrid breeding (Snowdon et al., 2015). Novel diversity from intraspecific, interspecific, and intergeneric sources is frequently used to breed for the improvement of resistance and heterosis (e.g., Snowdon et al., 2000; Li et al., 2004; Seyis et al., 2006; Rygulla et al., 2007; Zou et al., 2017); however, the genetic basis for secondary agronomic traits like seed vigor is generally considered only in the context of maintaining satisfactory performance without specific enrichment of elite genepools. Although substandard allele combinations for seed vigor have been largely eliminated from contemporary breeding material, breeders have not yet made full use of the genetic potential to further improve seed germination and vigor performance (Hatzig et al., 2015). Seed vigor and emergence have a complex inheritance and low heritability, being strongly influenced not only by sowing density, uniformity, and compensation propensity (Powell et al., 1991; TeKrony and Egli, 1991; Ellis, 1992; Hampton and Scott, 2011) but also strongly by both the seed ripening environment of the maternal plant and multiple temperature, water, and soil factors in the sowing environment (Hatzig et al., 2015). This makes it a particularly challenging trait for breeders.

Unintentional co-selection of secondary traits via linkage or pleiotropy can also influence breeding progress. Here we demonstrate that intense selection for specific seed qualities in winter oilseed rape breeding has had an inadvertent negative influence on seed germination performance. In a panel of 215 diverse winter oilseed rape varieties spanning over 50 years of breeding progress in winter-type rapeseed, we found that low seed EA content and reduced seed GSL content were significantly related with inferior seed germination. High-density genome-wide association studies revealed that this relationship is caused by linkage drag between important loci for seed quality and germination traits. Marker-assisted selection of suitable recombinants represents a simple method to further improve germination performance without negative effects on essential seed quality characters.

## Materials and Methods

### Data Acquisition and Pre-processing

A panel of 215 genetically diverse winter oilseed rape accessions was used for this study. The panel represents breeding progress spanning the transition in the 1970s and 1980s from rapeseed varieties with high EA and GSL content (++ quality), via intermediate forms (high EA, low GSL: +0, low EA, high GSL: 0+) into modern oilseed rape varieties with low EA and GSL content (00). Two different seed lots from the entire panel were produced by controlled self-pollination in two distinct environments during the growing seasons of 2010/2011 (SL2011) in Le Rheu (France, N48°6′3.95″, W1°47′43.38″) and 2011/2012 (SL2012) in Asendorf (Germany, N52°46′20.69″, E9°0′16.07″). On both locations, seeds were sown in field plots in early September. Normal location specific plant protection and fertilization measures were applied. Plants were harvested at full maturity and around 500 g seeds per genotype and production environment were collected for further analysis.

**FIGURE 1:**
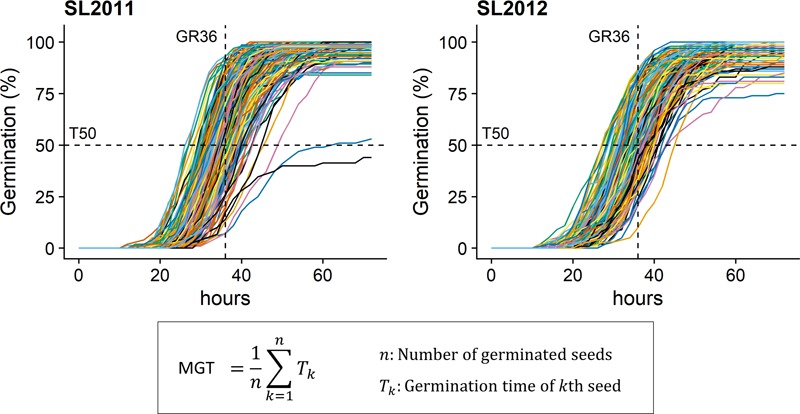
Germination kinetics for seeds from 215 diverse winter oilseed rape lines produced in 2011 (SL2011) and 2012 (SL2012). T50, time necessary to reach 50% of germination; GR36, Germination rate within 36 h; MGT: mean germination time.

MGT, GR36, and T50 were phenotyped in both seed lots under *in vitro* conditions (**Figure 1**) using the automated phenotyping platform of the French national seed testing station of the variety and seeds control group (Station Nationale d’Essais de Semences, Groupe d’Etude et de contrôle des Variétés et des Semences – GEVES, Angers, France). Detailed information about the phenotyping system is given by Demilly et al. (2014). In total, 100 seeds per genotype were analyzed in four replicates (25 seeds per replicate).

For classification of seed quality groups, the original cultivar declarations were taken into account and additionally confirmed by measurements of seed EA and GSL content in both seed lots, applying NIRS (Unity SpectraStar 2500, Brookfield, WI, United States). For differentiation between high and low GSL cultivars, a threshold of 25 μmol/g was chosen, according to the requirements of *Bundessortenamt Deutschland*. Because of the spectral similarities between oleic acid and EA, absolute values of EA are slightly overestimated by NIRS, and hence a threshold of 25% of total fatty acids was used to differentiate between high EA and low EA estimates. NIRS measurements were performed using standard procedures (Tillmann et al., 2000). The genotype panel comprised 125 accessions with 00 seed quality, 68 accessions with ++ quality, 20 accessions with 0+ quality (low EA, high GSL) and two accessions with +0 quality (high EA, low GSL). Accessions were classified according to the release period of their derivative cultivar as described by Bus et al. (2011). Correlations among traits were calculated by Pearson’s product moment correlation. Significant differences in germination performance among the quality groups were confirmed by one-way ANOVA and Student’s *t*-test. For testing of variance homogeneity, a Bartlett test was applied. Data distribution was evaluated by graphical display of data as well as by Shapiro–Wilk normality test.

### Genetic Relatedness and Genome-Wide Associations

The genotype panel was analyzed with the *Brassica* 60k Illumina^®^ R Infinium consortium SNP array (Edwards et al., 2013), according to standard procedures of the manufacturer. Pre-processing of marker data was done with the following settings: SNP markers with more than 20% missing calls across the panel were excluded. Furthermore, all individuals which had more than 20% missing calls across the genotype data were excluded. In order to incorporate rare alleles with potential effects on germination performance, only markers with a minor allele frequency ≤ 0.025 were excluded from analysis. A total of 192 genotypes and 22,169 SNPs met the chosen quality requirements. Genetic relatedness between genotypes was visualized in a principal coordinate analysis including the Euclidean modified Rodger’s distances using the free R package *SelectionTools* (downloadable^2^). For graphical representation, the first three principal coordinates were taken into account. Genetic differentiation was calculated in terms of overall *F*_ST_ among the seed quality subclusters using the software Genepop version 4.2.2 (Rousset, 2008). Calculation of genome-wide associations was done with the R package *GenABEL* (Aulchenko et al., 2007) applying a mixed model approximation including the first four principal coordinates as covariates to correct for population stratification. As shown by Price et al. (2010), this method is well suited for a correction of population structure, family structure, and cryptic relatedness between individuals in both sparsely and highly differentiated genotype panels.

Narrow-sense heritability for each trait was calculated separately for each seed lot, again using the R package GenABEL. Significance thresholds were defined by calculation of false discovery rates based on *p*-value statistics with the R package *fdrtool* (Strimmer, 2008). For MGT, seed EA, and GSL content, cutoff values between -log_10_(*p*-value) = 0.73 and 2.046 were calculated. Consequently, for all traits, only associations were taken into account which exceeded a value of -log_10_(*p*-value) = 2.1. Local LD decay was calculated as coefficient of correlation (*r*^2^) between the single SNP marker and visualized with R package LDheatmap (Shin et al., 2006). For physical localization of SNP markers, flanking sequences were blasted onto the *B. napus* Darmor-*bzh* reference genome sequence assembly (version 4.1; Chalhoub et al., 2014). SNPs were blasted using the following criteria: minimum overlap of 50 bp length, minimum identity of 95%, no sequence gaps. SNPs which failed across the entire genotype collection, as well as all locus non-specific SNPs for which more than one BLAST hit on the *B. napus* sequence was found, were excluded from further analyses. As heterozygous SNPs cannot be distinguished from multi-locus hemi-SNPs or false calls, heterozygous calls were treated as missing values. Genes near to trait-associated markers were characterized with the tool Blast2GO (Conesa et al., 2005) using default settings. Confidence regions harboring potential candidate genes were defined as the region between two markers next to an associated marker. When flanking markers were in strong LD (*r*^2^ > 0.4) with an associated marker, regions were defined as LD blocks and the whole LD block was taken into account for candidate gene disclosure. Candidates were selected based on GO terms related to either seed germination, EA biosynthesis, or GSL synthesis taking into account all associated metabolic processes as well.

## Results

### Phenotype Performances and Trait Correlations

**FIGURE 2:**
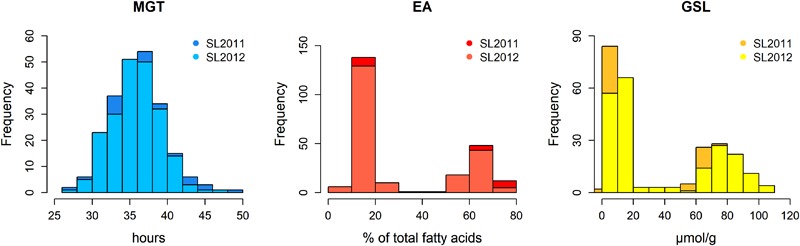
Histograms showing the phenotypic distributions of mean germination time (MGT), seed erucic acid content (EA), and seed glucosinolate content (GSL) in a diversity panel comprising 215 winter oilseed rape genotypes produced in 2010/11 (SL2011) and 2011/12 (SL2012).

The germination traits MGT, T50, and GR36 showed strong inter-trait correlations (|*r*| > 0.93; **Table 1**). Furthermore, in both seed lots, germination performance showed significant correlations to both EA content and GSL content, particularly in SL2011 (0.42 > |*r*| > 0.33) but also in SL2012 (0.39 > |*r*| > 0.29). As strongest correlations to EA and GSL content were observed in both seed lots for MGT, this parameter was chosen for further investigations aiming to disclose possible genetic relations between germination performance and seed quality. In contrast, no or only weak correlations were observed between germination performance and other acquired seed compounds like oil content, protein content, oleic acid content, and linoleic acid content (**Supplementary Figure S1**). The two seed lots showed significant correlations with each other both within and among the different germination traits (0.66 > |*r*| > 0.60). For germination performance in terms of MGT, a Gaussian distribution was observed (**Figure 2**). Similar distributions were also observed for GR36 and T50 (data not shown). As expected, EA and GSL content deviated clearly from a normal distribution, reflecting the inheritance of the low EA trait by two recessive gene mutations (Ecke et al., 1995; Fourmann et al., 1998) and low seed GSL by three major QTL (Uzunova et al., 1995). As strongest correlations to EA and GSL content were observed in both seed lots for MGT, this parameter was chosen for further investigations aiming to disclose possible genetic relations between germination performance and seed quality.

**Table 1:** Correlation coefficients (*r*) between germination performance traits and seed quality traits measured in two seed lots produced in 2011 and 2012 from an winter oilseed rape diversity panel (*n* = 215).

		2011					2012				
		GR36	MGT	T50	EA	GSL	GR36	MGT	T50	EA	GSL
**2011**	**MGT**	-0.97	^∗∗∗^	^∗∗∗^	^∗∗∗^	^∗∗∗^	^∗∗∗^	^∗∗∗^	^∗∗∗^	^∗∗∗^	^∗∗∗^
	**T50**	-0.93	0.94	^∗∗∗^	^∗∗∗^	^∗∗∗^	^∗∗∗^	^∗∗∗^	^∗∗∗^	^∗∗∗^	^∗∗∗^
	**EA**	0.36	-0.40	-0.33	^∗∗∗^	^∗∗∗^	^∗∗∗^	^∗∗∗^	^∗∗∗^	^∗∗∗^	^∗∗∗^
	**GSL**	0.38	-0.42	-0.35	0.77	^∗∗∗^	^∗∗∗^	^∗∗∗^	^∗∗∗^	^∗∗∗^	^∗∗∗^
**2012**	**GR36**	0.64	-0.65	-0.62	0.32	0.35	^∗∗∗^	^∗∗∗^	^∗∗∗^	^∗∗∗^	^∗∗∗^
	**MGT**	-0.61	0.64	0.60	-0.35	-0.35	-0.95	^∗∗∗^	^∗∗∗^	^∗∗∗^	^∗∗∗^
	**T50**	-0.64	0.66	0.64	-0.30	-0.33	-0.97	0.96	^∗∗∗^	^∗∗∗^	^∗∗∗^
	**EA**	0.37	-0.40	-0.34	0.99	0.77	0.31	-0.33	-0.29	^∗∗∗^	^∗∗∗^
	**GSL**	0.40	-0.44	-0.37	0.77	0.98	0.38	-0.39	-0.36	0.77	^∗∗∗^

*MGT, mean germination time; GR36, germination rate within 36 h; T50, time to reach 50% of germination; EA, seed erucic acid content; GSL, seed glucosinolate content. Significant differences at ^∗∗∗^*p* < 0.001.*

**FIGURE 3:**
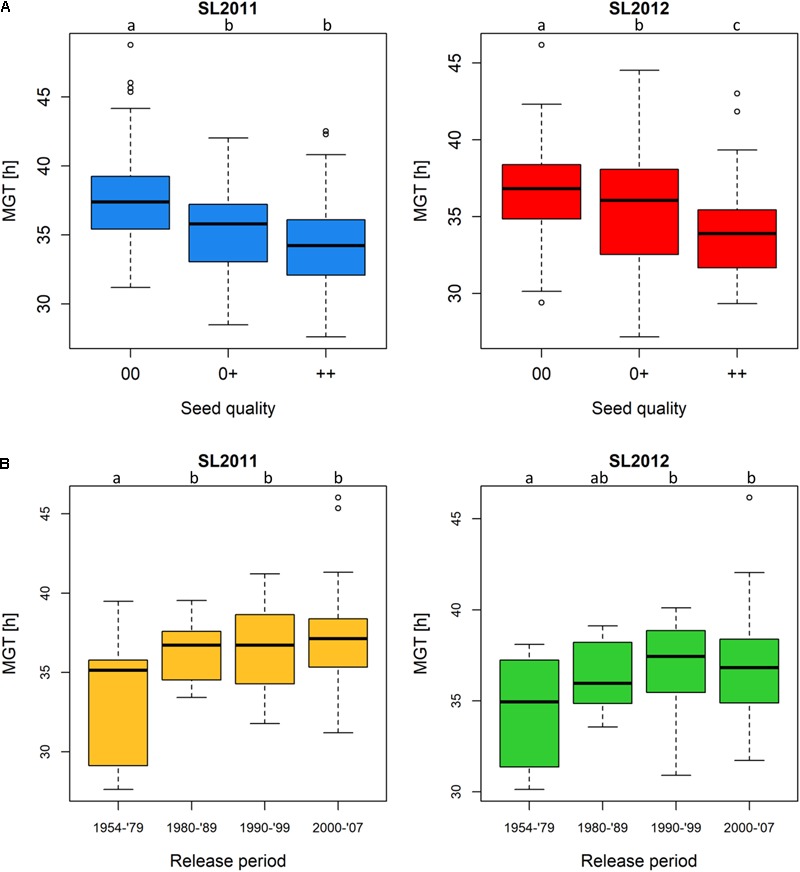
Boxplots showing germination performance in a diverse set of winter oilseed rape genotypes. **(A)** Mean germination time (MGT) of genotypes classified by seed quality: 00, low erucic acid, low glucosinolate content; 0+, low erucic acid, high glucosinolate content; ++, high erucic acid, high glucosinolate content. **(B)** MGT of genotypes classified by their release periods: 1954–1979 (comprising ++ and 0+ genotypes solely), 1980–1989 (comprising ++, 0+ and 00 genotypes), 1990–1999, and 2000–2007 (00 genotypes only). Significant differences were calculated at a level of *p* < 0.1. Significance groups were indicated as letters.

Assigning all 215 genotypes to their quality groups revealed that, in both seed lots, accessions with 00 quality showed a poorer germination performance compared to accessions with 0+ and ++ quality, respectively (**Figure 3A**). Since only two genotypes showed +0 quality, this quality group was underrepresented and not taken into consideration. In both seed lots, MGT was significantly higher in 00 than in the 0+ and ++ groups, respectively, while seed lot SL2012 (but not SL2011) showed significantly higher MGT in 0+ than ++ accessions. In accordance with the changes in seed quality of released varieties during the past three decades, accessions derived from cultivars released earlier than 1980 (++ and 0+) showed a significantly better germination performance than accessions derived from cultivars released after 1980, which include all 00 cultivars in the panel (**Figure 3B**). After 1979, no significant differences were observed between the different release periods in either seed lot, supporting a hypothesis that the difference in germination is attributable to a direct association with seed quality rather than a general and ongoing consequence of selective breeding over time. Accessions derived from cultivars released between 1954 and 1979 showed mean MGT values of 33.4 and 34.4 h for SL2011 and SL2012, respectively, while mean MGT values for the genotypes released between 1980 and 2007 were 36.6 h (SL2011) and 36.7 h (SL2012).

### Genetic Differentiation and Genome-Wide Associations

**FIGURE 4:**
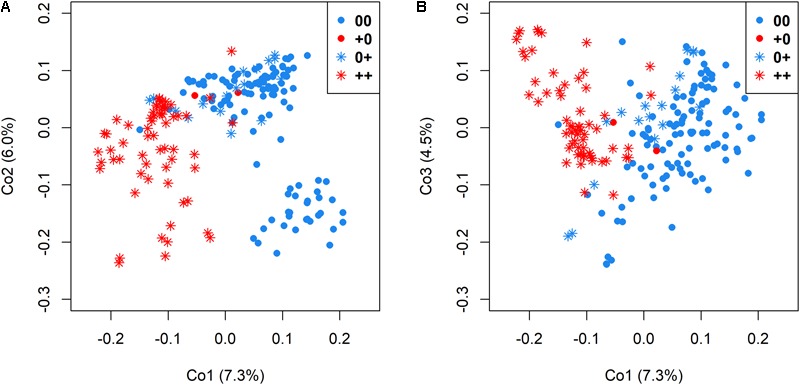
Genetic relatedness of 215 genetically diverse winter-rapeseed lines shown in a principal coordinate analysis regarding the first and second coordinate **(A)** and the first and third coordinate **(B)**. Different seed qualities were represented by different colors and symbols.

Genetic relatedness among genotypes in relationship to the different seed quality groups was visualized by cluster analysis among accessions with 00, ++, 0+, and +0 quality, respectively. A clear differentiation between low EA and high EA accessions was revealed by the first principle coordinates, which explained 7.3% and 6.0% of total genetic variance (**Figure 4A**), whereas differentiation between accessions with low and high GSL was possible by also considering principle coordinate 3 (which explained 4.5% of the overall variation in the panel, **Figure 4B**).

A moderate genetic differentiation (*F*_ST_ = 0.10) was calculated between the two sub-clusters differing in EA content, whereby low GSL and high GSL genotypes were somewhat less clearly differentiation (*F*_ST_ = 0.08). This reflects the difference between the simple, bigenic inheritance for EA, and the more complex oligogenic inheritance for GSL. In contrast, the two groups representing accessions with 00 and 0+ quality were only poorly differentiated (*F*_ST_ = 0.04), whereas a higher differentiation (*F*_ST_ = 0.09) was calculated between accessions with 0+ and ++ quality and the highest differentiation (*F*_ST_ = 0.11) between accessions with 00 and ++ quality.

Very high heritabilities were calculated for GSL content, with values of *h*^2^ = 0.96 and *h*^2^ = 0.99 for SL2011 and SL2012, respectively. Similar values were calculated for EA content, with *h*^2^ = 0.86 (SL2011) and *h*^2^ = 0.85 (SL2012), whereby this somewhat lower value may reflect inaccuracies in NIRS estimates of very low EA concentrations. For MGT, heritabilities of *h*^2^ = 0.75 (SL2011) and *h*^2^ = 0.82 (SL2012) were observed.

**FIGURE 5:**
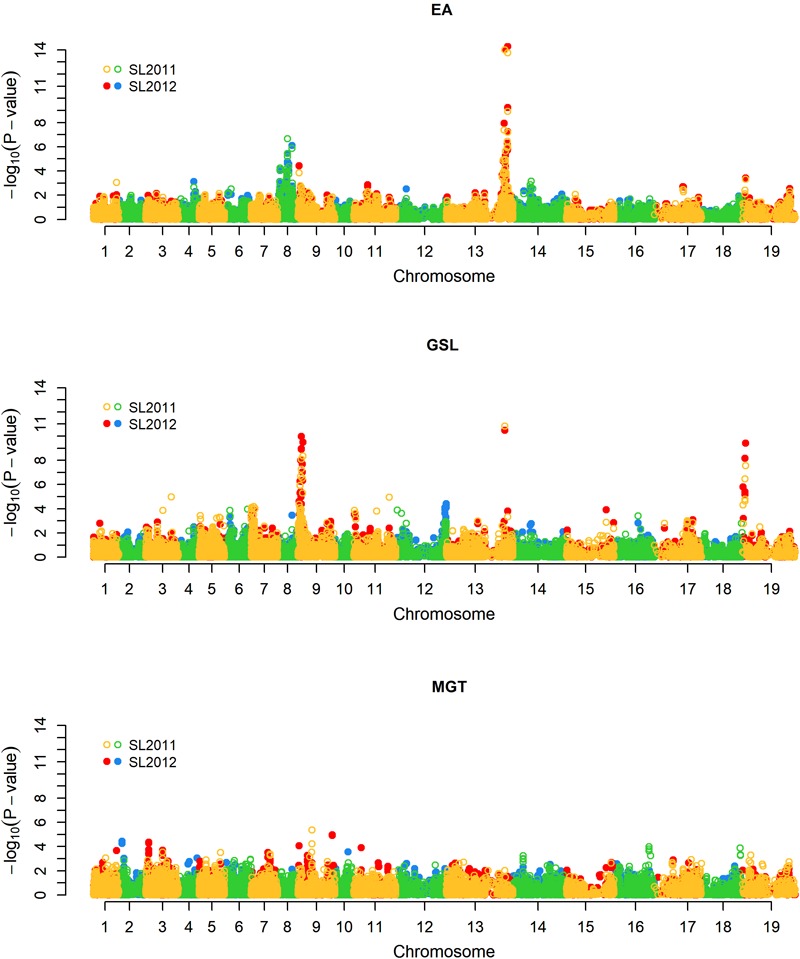
Manhattan plot describing genome-wide marker-trait associations for the traits seed erucic acid content, seed glucosinolate content, and mean germination time. Yellow and green dots represent associations calculated for seed lot SL2011. Red and blue dots represent associations calculated for seed lot SL2012. EA: Seed erucic acid content, GSL: Seed glucosinolate content, MGT: Mean germination time.

**FIGURE 6:**
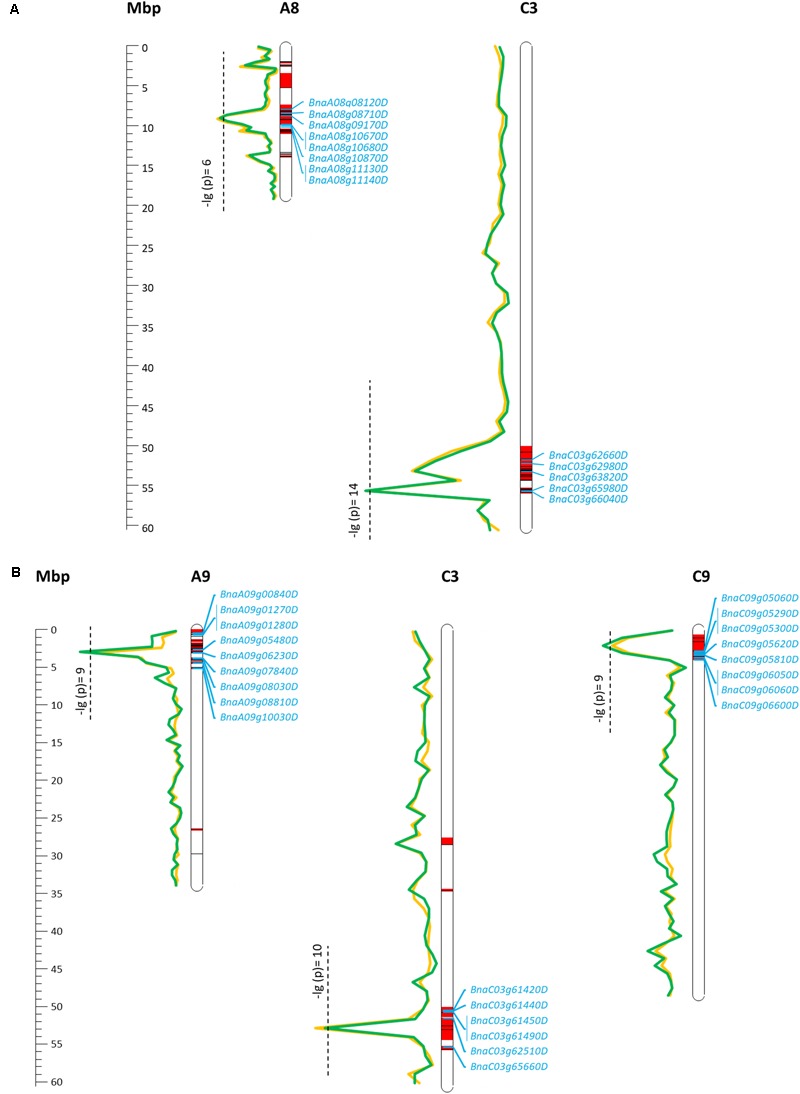
Main marker–trait associations calculated for seed erucic acid content **(A)** and seed glucosinolate content **(B)** within a diverse set of winter oilseed rape genotypes. Calculated -lg(*p*) values for the two observed seed lots were represented by a green (SL2011) and a yellow line (SL2012). Confidence intervals harboring associated SNP markers and SNP markers which are in LD (*r*^2^ > 0.4) to an associated marker are shown in red. Candidate genes enclosed by the confidence regions are shown in blue.

For EA content, GSL content, and MGT, diverse trait-associated genomic regions could be identified by mixed model approximation (**Figure 5**). A complete list of all significant associations is provided in **Supplementary Table S1**. As expected, strongest associations for EA content were localized on chromosomes A08 and C03 (**Figure 6A**) corresponding to the known positions of the two *Bna.FAE1* homologs (Fourmann et al., 1998). The *Bna.FAE1* copy on chromosome A8 (*BnaA08g11130D*) lies 886.1 kb from peak marker Bn-A08-p11458466, for which -log_10_-*p*-values of 6.66 (2011) and 5.42 (2012) were calculated. On chromosome C03, *Bna.FAE1* (*BnaC03g65980D*) is located 25.6 kb away from marker Bn-scaff_15794_3-p83984, which showed the highest -log_10_-*p*-values of 14.29 and 13.75 in seed lots SL2011 and SL2012, respectively.

For seed GSL content, highly significant QTL were located on chromosomes A09, C03, and C09 with -log_10_-*p*-values of 9.97 (SL2011), 10.83 (SL2012), and 9.42 (SL2011), respectively (**Figure 6B**). The peaks for the major QTL for seed GSL content lie near to the GSL biosynthesis gene *HIGH-ALIPHATIC GLUCOSINOALTE 1* (*Bna.HAG1/MYB28*: Hirai et al., 2006; Gigolashvili et al., 2007; Harper et al., 2012) on chromosome C09 (*BnaC09g05290D*) and to homeologs of the transcription factor *Bna.MYB34* (Frerigmann and Gigolashvili, 2014; Miao et al., 2016) on chromosomes A09 (*BnaA09g05480D*) and C09 (*BnaC09g05060D*), respectively.

To investigate possible pleiotropy or linkage in repulsion between seed quality and germination traits, we investigated co-localization of significant QTL for GSL and EA with positions of QTL for MGT. Neither of the two major loci controlling EA showed a relationship to germination; however, one minor QTL for MGT on chromosome A09 (close to but not overlapping the major QTL for GSL), co-localized with significant but minor QTL for both EA content and MGT (**Figure 5B**). This suggested either potential pleiotropy or close linkage of minor factors influencing all three traits.

**FIGURE 7:**
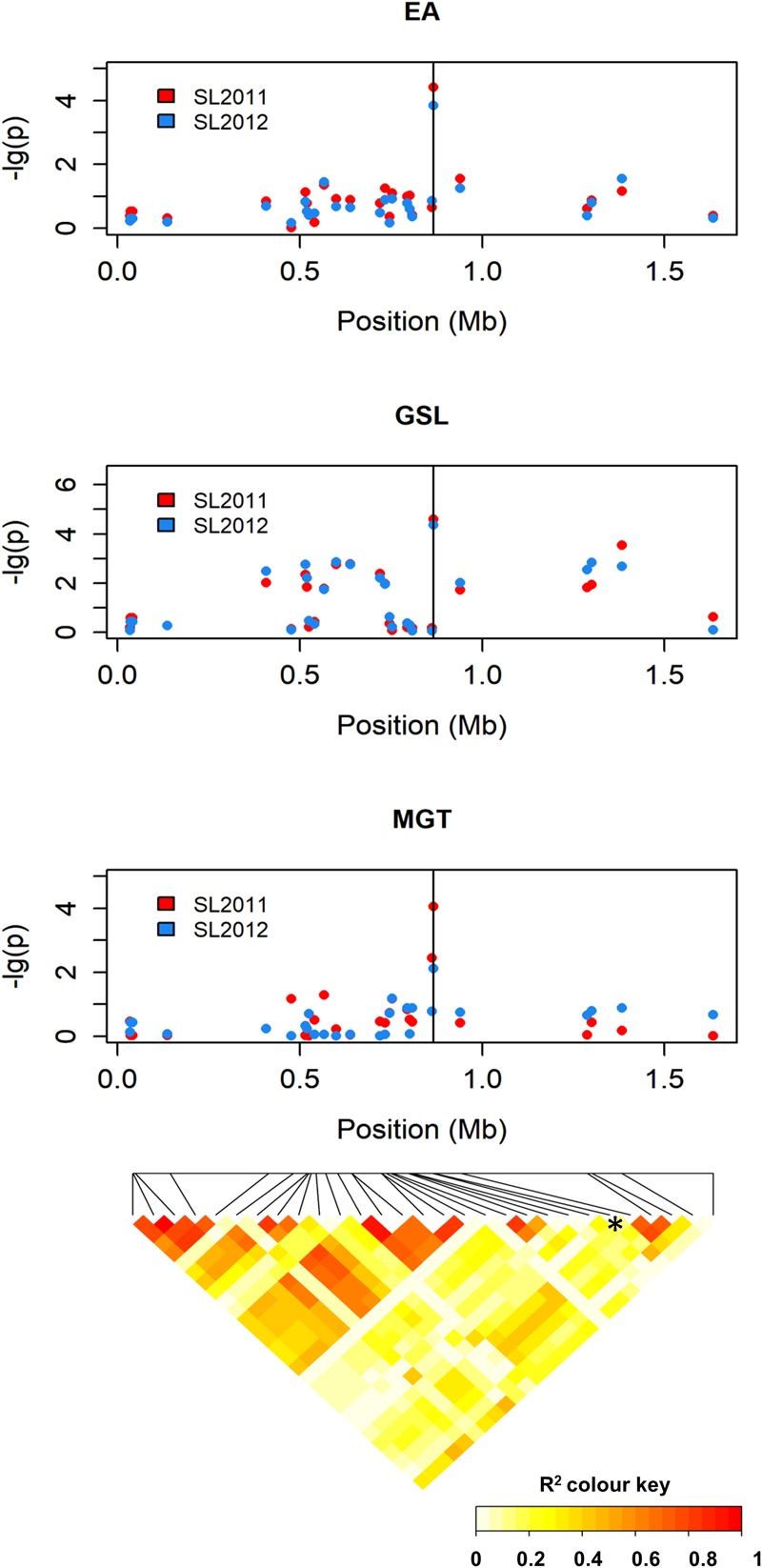
Manhattan plot describing genome-wide marker–trait associations for the traits seed erucic acid content, seed glucosinolate content, and mean germination time around peak marker Bn-A01-p26914512 on chromosome A9 and heatmap showing the corresponding chromosome-wide correlations (*r*^2^) between SNP markers.

The SNP marker with the strongest association to MGT (Bn-A01-p26914512; *p*-value = 4.06) exhibited only marginal LD with the surrounding markers (**Figure 7**), both when LD was calculated over the entire population and within individual seed quality groups. Therefore, no haplotypes could be defined by including neighboring SNP markers, and further inspection focused on the single peak marker. The chromosomal region delineated by marker Bn-A01-p26914512 harbored a total of 26 genes (**Supplementary Table S2**). GO analysis revealed no genes with known functions related to EA or GSL biosynthesis; however, two genes (*BnaA09g01570D and BnaA09g01640D*) exhibit annotations related to seed germination and are thus regarded as potential candidates for this QTL.

Allele frequencies and allelic effects for the SNP marker Bn-A01-p26914512 were compared across the three different seed quality groups (00, 0+, and ++) to detect potential bias due to linkage with the nearby major QTL peak for GSL, along with possible pleiotropic effects due to the overlap with minor QTL for GSL and EA. Because segmental deletions caused by widespread homoeologous exchanges (Samans et al., 2017) are frequently associated with trait variation in *B. napus* (Qian et al., 2016; Schiessl et al., 2017; Stein et al., 2017), we also considered the possibility that failed calls for SNP Bn-A01-p26914512 are in fact deletions that can show trait associations independently of the two SNP alleles (Grandke et al., 2016; Mason et al., 2017). The SNP assay for Bn-A01-p26914512 failed to show a signal in 10%, 10%, and 26% of the 00, 0+, and ++ genotypes, respectively.

**FIGURE 8:**
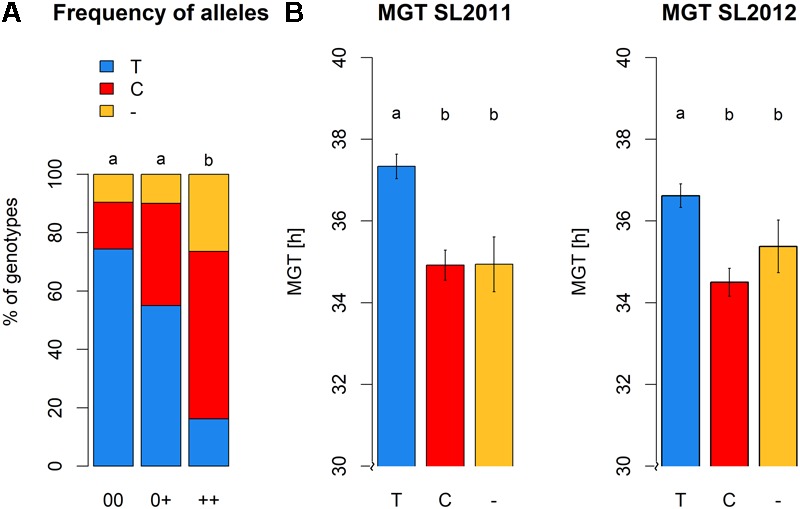
**(A)** Allele frequencies of peak marker Bn-A01-p26914512 within the different seed quality groups. 00, low erucic acid, low glucosinolate content; 0+, low erucic acid, high glucosinolate content; ++, high erucic acid, high glucosinoate content. **(B)** Mean germination time of genotypes grouped with regard to their marker allele predominant at locus Bn-A01-p26914512, calculated for seed lot 2011 and seed lot 2012.

Significant differences were detected between the three seed quality groups for the frequencies of SNP alleles C or T and the deletion (null allele) at locus Bn-A01-p26914512 (**Figure 8**). Furthermore, these allelic differences associated with a clear shift in germination time in the different seed quality groups (**Figure 8**). Specifically, the SNP allele (T) associated with slower germination was significantly more prevalent in cultivars with low EA content (00 and 0+ seed quality). Marker allele T was present in nearly 75% of all genotypes with 00 quality, but only in 55% of the 0+ and 16% of the ++ quality groups, respectively. Conversely, marker allele C was present in 57% of the ++ genotypes, but only 35% of 0+ and 16% of 00 genotypes.

**FIGURE 9:**
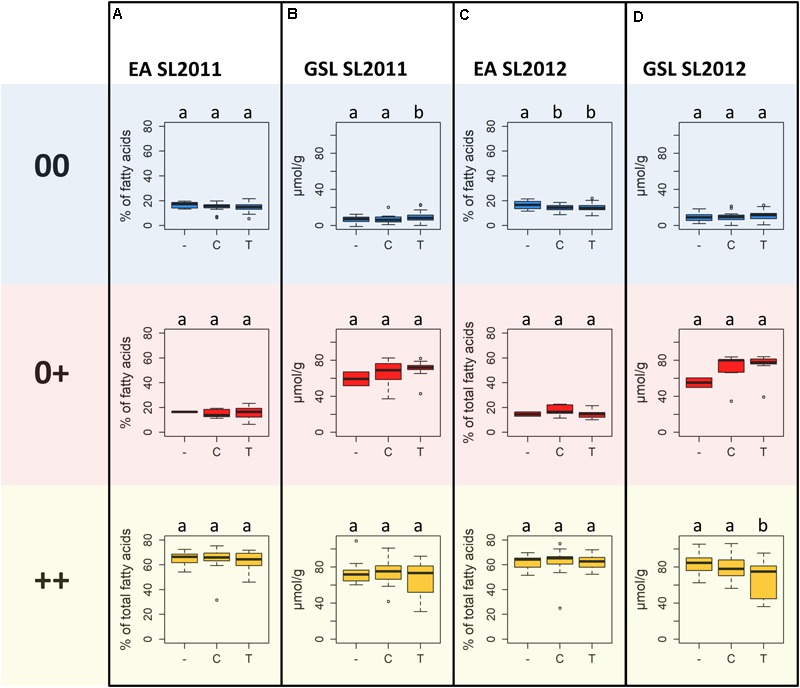
Boxplots showing **(A)** seed erucic acid (EA) content of seed lot SL2011 and **(B)** seed glucosinolate (GSL) content of seed lot SL2011, and **(C)** seed EA content of seed lot SL2012 and **(D)** seed GSL content of seed lot SL2012 dependent on the predominant marker allele of peak marker Bn-A01-p26914512, determined for the different seed quality groups (00, low erucic acid, low glucosinolate content; 0+, low erucic acid, high glucosinolate content; ++, high erucic acid, high glucosinoate content).

Highest MGT values for both seed lots were observed for individuals carrying allele T at SNP Bn-A01-p26914512. These values were significantly higher than the MGT values of individuals with allele C or failed calls, suggesting that the failed calls are not (only) random amplification failures. No significant difference in MGT was observed between individuals carrying allele C and individuals with a putative deletion of Bn-A01-p26914512. The different alleles and the putative deletion of Bn-A01-p26914512 showed no reproducible, significant effect on seed quality within each respective seed quality group (**Figure 9**). Among genotypes with 00 seed quality, the only significant effect was found for EA in SL2012 (**Figure 9C**) where an absence of marker Bn-A01-p26914512 appeared to associate with a slight (up to 2%) increase in seed EA content. However, this effect could not be confirmed by the second seed lot (**Figure 9A**). For GSL content, contrary effects were found for the allele T, which appeared to associate with a slight increase in GSL content in 00 genotypes in SL2011 (**Figure 9B**), whereas a slight reduction in GSL content was observed in ++ genotypes in SL2012 (**Figure 9D**). Again, however, neither effect could be confirmed by the second seed lot.

## Discussion

Enormous progress has been made during the brief breeding history of rapeseed in improvement of seed quality, as a primary prerequisite for large-scale utilization and international production of the crop, and more recently in key crop production characteristics with a focus on seed yield. Due to the major focus of seed quality and yield, improvement of secondary agronomic traits like germination often plays a secondary role. Strong natural and artificial selection acting against individuals with very poor germination and vigor has restricted phenotypic variability among cultivated winter-type forms of *B. napus* (Hatzig et al., 2015). Nevertheless, utilization of exotic germplasm is still an important breeding task to reinstate diversity for heterotic vigor and disease resistances. Less reliable germination in exotic or non-adapted materials, for example, synthetic *B. napus* used for enrichment of depleted breeding pools (Snowdon et al., 2015) or enhancement of resistance (Rygulla et al., 2007; Mei et al., 2015), presents a considerable challenge for breeders due to the limited heritability of germination and emergence traits.

Here we found evidence that the significant correlation between seed quality and germination characteristics may derive from negative pleiotropic effects of minor loci with opposite effects [positive vs. negative on seed quality (GSL and EA content) and germination performance]. Further studies are required to determine whether the two candidate genes for MGT which lie in the QTL interval have a direct, negative pleiotropic effect on GSL and EA, or whether the trait associations between seed quality and germination time are caused by linkage in repulsion of other nearby, unidentified genes. The gene *BnaA09g01570D* is an ortholog of the *Arabidopsis thaliana* gene *EMBRYO DEFECTIVE 2770* (*EMB2770*; *At4g03430*), which is proposed to act as splicing factor involved in the RNA-directed methylation pathway (Dou et al., 2013). Mutations in this gene cause a defective embryo development during the transition phase (Meinke et al., 2008) and thus early developmental arrest. A further consequence is hypersensitivity to ABA, leading to impaired germination and seedling root growth (Lee et al., 2006). A second functional candidate in the QTL interval is *BnaA09g01640D*, an ortholog of the *A. thaliana* gene *TPC1* (AT4G03560). *TPC1* encodes a depolarization-activated Ca^2+^ channel protein which mediates calcium influx in numerous plant tissues and developmental stages. According to Peiter et al. (2005), *TPC1* appears to play an essential role in the ABA-dependent regulation of seed germination. Greco et al. (2012) found that plant TPC channels might be modulated by polyunsaturated fatty acids, providing a potential link between *TPC1* and the seed fatty acid composition; however, this potential mode of pleiotropic interaction between EA content and germination capacity requires deeper study on a molecular level.

Interestingly, cultivars released prior to 1980 generally showed superior MGT to cultivars released later (**Figure 3B**). This coincides with the registration of the first 00 cultivar in Germany in 1981. The deterioration in germination quality in modern cultivars is in contrast to breeding progress for yield potential, which accelerated strongly after the introduction of 00 cultivars due to intensified cultivation and breeding (Stahl et al., 2017).

Our results suggest that the reduction in germination performance may be due to inadvertent co-selection of genetic variants associated with 00 seed quality that have a negative influence on germination. Further studies are required to reveal whether this relationship is due to negative pleiotropic effects of one or more genes within the minor QTL represented by SNP marker Bn-A01-p26914512, or by linkage in repulsion between germination factors (for example, the candidate genes described above) and unknown genes with a minor effect on EA and/or GSL metabolism. If the latter is the case, then the apparently low LD levels near this QTL suggest a high recombination potential to disrupt undesired linkage. On the other hand, the small effect of the QTL on the two seed quality traits raises the possibility to improve germination by selection for suitable haplotypes that confer enhanced germination, with only a minor compromise in EA and GSL content provided that the major-effect loci for these traits are represented. Indeed, 32 out of 125 00 cultivars in our test panel combine 00 quality with low MGT (in association with the desirable C or null alleles at SNP locus Bn-A01-p26914512). This suggests either that the linkage in repulsion at this locus has been broken in these genotypes or that their seed quality is conferred solely by the major QTL without an influence of the minor QTL on chromosome A09.

Different environmental factors influence seed GSL and EA content, including plant water supply (Bouchereau et al., 1996; Champolivier and Merrien, 1996; Jensen et al., 1996) and fertilization (Asare and Scarisbrick, 1995). Nevertheless, heritability is high for both traits due to the major contribution of dominant genetic regulators in the underlying biosynthetic pathways (Shi et al., 2003; Verkerk et al., 2009). On the other hand, heritability for seed germination performance in *B. napus* shows a broad range depending largely on the degree of environmental stress (Acharya et al., 1983; Bettey et al., 2000; Hatzig et al., 2015). Our results demonstrate that narrow-sense heritability is comparatively high in a genotype panel with a broad phenotypic variance, particularly when germination is assayed under optimal *in vitro* conditions. Under field conditions, heritability of germination and emergence rate is lower, presenting difficulties for phenotypic selection.

Since the process of seed germination is under complex polygenic control (Bettey et al., 2000; Hatzig et al., 2015), we did not expect single genetic factors to have a major influence on trait performance. In contrast to this expectation, the observed association of allelic differences at SNP Bn-A01-p26914512 with a clear shift in germination time (**Figure 8**) suggests that intensive selection for low EA content caused inadvertent co-selection of a QTL causing reduced germination speed. This may be due to pleiotropy or linkage in repulsion; however, in either case, the existence of 00-quality individuals where this negative relationship is no longer observed provides a basis for future selection to avoid this problem. This QTL has only a minor effect on the major seed quality traits which is not relevant in comparison to the essential effects of the major EA and GSL loci. Hence, marker-assisted selection of positive alleles for MGT at locus Bn-A01-p26914512 can help breeders to maintain maximal germination capacity in new 00-quality oilseed rape cultivars.

## Author Contributions

SH and RS designed the study, interpreted the results, and wrote the manuscript. RS, NN, AA, and GL developed the diversity panel and generated seed lots for the phenotypic analysis. SD and M-HW performed the automated germination phenotyping, and FB generated the genome-wide SNP data. SH performed the NIRS and GWAS analysis. All authors corrected and approved the final version.

## Conflict of Interest Statement

The authors declare that the research was conducted in the absence of any commercial or financial relationships that could be construed as a potential conflict of interest.

## Footnotes

1 www.faostat.org

2 http://www.uni-giessen.de/fbz/fb09/institute/pflbz2/population-genetics/software

## Supplementary Material

FIGURE S1 Correlation coefficients (*r*) between germination performance traits and seed oil content, seed protein content, seed oleic acid content, and seed linolenic acid content measured in two seed lots produced in 2011 and 2012. MGT, mean germination time; GR36, germination rate within 36 h; T50, time to reach 50% of germination. Significant differences at *p* < 0.001^∗∗∗^, *p* < 0.01^∗∗^, and *p* < 0.05^∗^.

TABLE S1 List of all SNP markers which are significantly associated with mean germination time (MGT), seed erucic acid (EA) content, and seed glucosinolate (GSL) content along with their corresponding -log_10_-*p*-values calculated for seed lot 2011 [-log10(*P*)_2011] and seed lot 2012 [-log10(*P*)_2012]. A1 and A2, alternative alleles of SNP marker.

TABLE S2 List of genes, associated with QTL marker Bn-A01-p26914512 on chromosome A9. Gene-ID, name of gene; Start: physical start position of gene in bp; Stop: physical stop position of gene in bp.

## Abbreviations

00: low erucic acid, low glucosinolate
0+: low erucic acid, high glucosinolate
+0: high erucic acid, low glucosinolate
++: high erucic acid, high glucosinolate
ABA: abscisic acid
*Bna.FAE1*: *Brassica napus FATTY ACID ELONGASE 1*
*Bna.HAG1*: *B. napus HIGH-ALIPHATIC GLUCOSINOALTE 1*
*Bna.MYB*: *B. napus transcription factor MYB 34*
EA: erucic acid
*EMB2770*: e*mbryo defective 2270*
*F*_ST_: fixation index
GO: gene ontology
GR36: germination rate within 36 h
GSL: glucosinolate
LD: linkage disequilibrium
MGT: mean germination time
NIRS: near-infrared reflectance spectroscopy
QTL: quantitative trait locus
SL2011: seed lot produced in 2011
SL2012: seed lot produced in 2012
SNP: single nucleotide polymorphism
T50: time to reach 50% of germination
TPC1: two-pore channel 1
