# Supplementary figures and images for: Hidden Effects of Seed Quality Breeding on Germination in Oilseed Rape (Brassica napus L.)
Source: Front Plant Sci. 2018 Apr 3;9:419. doi: 10.3389/fpls.2018.00419 (PMC5891602; doi:10.3389/fpls.2018.00419)

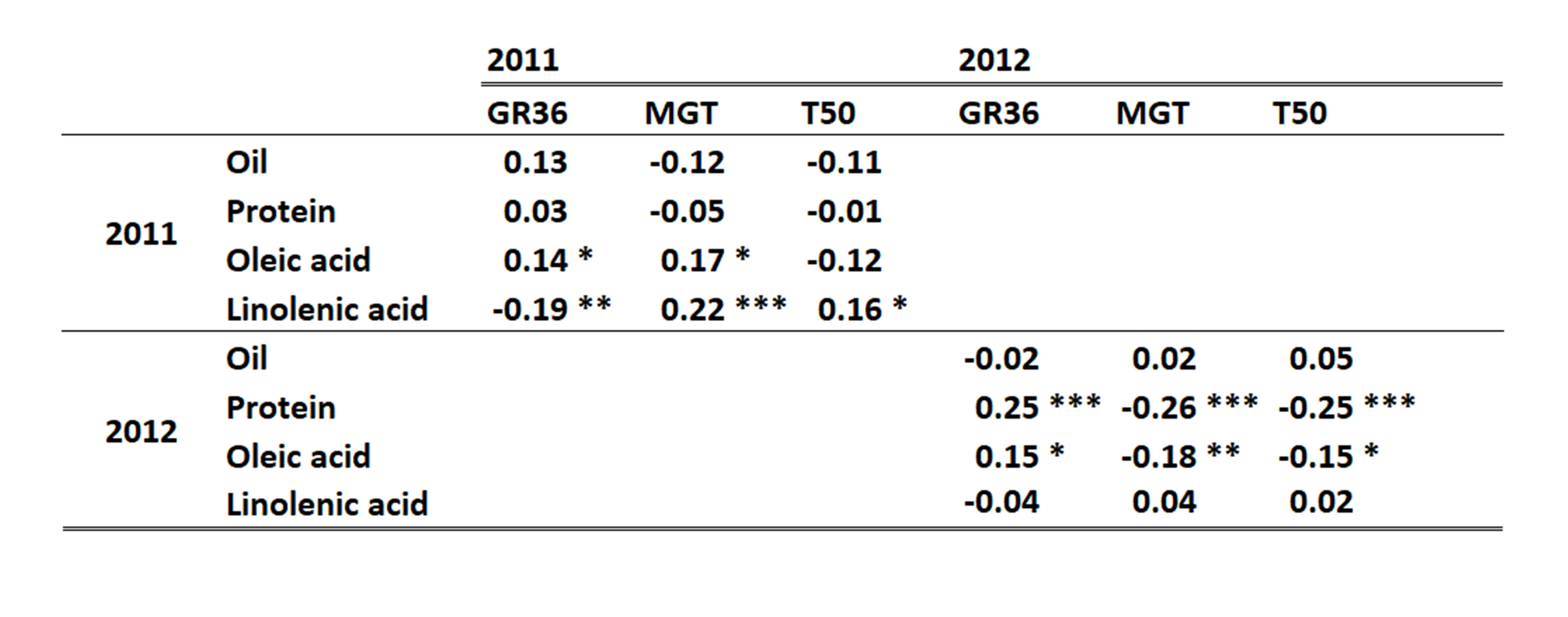

Supplement: FIGURE S1 — Correlation coefficients (r) between germination performance traits and seed oil content, seed protein content, seed oleic acid content, and seed linolenic acid content measured in two seed lots produced in 2011 and 2012. MGT, mean germination time; GR36, germination rate within 36 h; T50, time to reach 50% of germination. Significant differences at p < 0.001∗∗∗, p < 0.01∗∗, and p < 0.05∗. [file Image_1.TIF]
